# Cardiac shock wave therapy promotes arteriogenesis of coronary micrangium, and ILK is involved in the biomechanical effects by proteomic analysis

**DOI:** 10.1038/s41598-018-19393-z

**Published:** 2018-01-29

**Authors:** Wenhui Yang, Yan He, Lulu Gan, Fan Zhang, Baotong Hua, Ping Yang, Juan Liu, Li Yang, Tao Guo

**Affiliations:** 1 Department of Geriatrics, Yan’an Affiliated Hospital of Kunming Medical University, Kunming, China; 2 Key Laboratory of Cardiovascular Disease of Yunnan Province, Kunming, China; 3 Department of Cardiology, 1st Affiliated Hospital of Kunming Medical University, Kunming, China; 4 Department of Ultrasound, 2nd Affiliated Hospital of Kunming Medical University, Kunming, China

## Abstract

Cardiac Shock Wave Therapy (CSWT) improves myocardial perfusion and ameliorates cardiac remodeling after acute myocardial infarction (AMI), but the precise mechanisms remain obscure. Herein, we have applied CSWT to a rat model of AMI to demonstrate the arteriogenesis of coronary micrangium and protein expression changes in ischemic myocardium after CSWT. Four weeks after CSWT, the fraction shortening of rats was improved greatly and the cardiomyocyte apoptosis index was significantly lower than the AMI group (P < 0.05). Besides, the fibrotic area was markedly decreased in the CSWT group. In the infarction border zone, the thickness of smooth muscle layer was expanded apparently after CSWT. Label-free quantitative proteomic analysis and bioinformatics analysis revealed that the differentially expressed proteins were largely enriched in the focal adhesion signaling pathway. And integrin linked kinase (ILK) may be a key factor contributed to arteriogenesis of coronary micrangium during CSWT. In conclusion, non-invasive cardiac shock wave could promote arteriogenesis of coronary micrangium and alleviate myocardial apoptosis and fibrosis after AMI. Furthermore, focal adhesion signaling pathway may have a central role in the related signal network and ILK was closely related to the arteriogenesis of coronary micrangium during CSWT.

## Introduction

The number of patients with ischemic heart disease (IHD) is rapidly increasing worldwide and post-infarction heart failure remains the major cause of morbidity and mortality. Currently, classic revascularization methods such as percutaneous coronary intervention (PCI) and coronary artery bypass grafting (CABG) can effectively relieve the stenosis or occlusion of epicardial coronary artery. However, whether the distal coronary microcirculation is subsequently improved is not yet clear. A series of clinical trials have found the combination treatment of epicardial coronary revascularization and medication can improve clinical symptoms but cannot prevent the occurrence of myocardial infarction and death^1–3^. Part of that is because the dysfunction of coronary microcirculation, which is the last station of supplying the myocardial blood and oxygen and transporting metabolites, has not been appreciated seriously. Dysfunction of coronary microcirculation could seriously impair myocardial perfusion^4–6^. Relieving the epicardial coronary stenosis is a prerequisite of revascularization, but the key point to reduce myocardial ischemia is by improving of coronary microcirculation.

Based on the consideration that angiogenesis might reverse the pathophysiologic process that leads to IHD^7^, many novel approaches have emerged to improve the coronary microcirculation involving transmyocardial revascularization (TMR), stem cell transplantation and intracoronary administration of cytokine which are still at a preclinical stage^8,9^. However, cardiac shock wave therapy (CSWT), as a new therapy inducing angiogenesis in the border zone of infarcted myocardium, has drawn greater attention. We have previously demonstrated that CSWT induced the proliferation and differentiation of endothelial progenitor cells^10^ and human umbilical vein endothelial cells (HUVECs)^11^ in patients with IHD. Additionally, we observed significant up-regulation of mRNA levels of vascular endothelial growth factor (VEGF) and its receptors, which have previously been suggested to induce angiogenesis in ischemic tissue after shock-wave treatment^12^. Angiogenesis describes the growth of endothelial sprouts from preexisting postcapillary venules to form capillary networks. In angiogenesis, only small capillaries are formed which are ineffective at restoring robust perfusion to ischemic tissue. In contrast, arteriogenesis refers to formation of functional, larger collateral arteries from pre-existing arteriolar anastomoses^13^. Shear stress is an important trigger to promote arteriogenesis^14^. However, whether the shear stress produced by CWST could promote arteriogenesis has not yet been studied and the time course changes of molecular in myocardium tissues has not been gained.

To further investigate the biological effect and mechanisms of CSWT, we built Sprague-Dawley (SD) rat model of acute myocardial infarction and assessed the efficacy of CSWT in promoting arteriogenesis of coronary micrangium. Label-free quantitative proteomic technology and bioinformatics analysis were applied for analyzing the key regulators of CSWT on rat myocardium and coronary micrangium in the biological process. This study therefore represents the basis for future clinical application of CSWT in patients with IHD.

## Results

### CSWT improved cardiac function of rats with myocardial infarction

**Figure 1:**
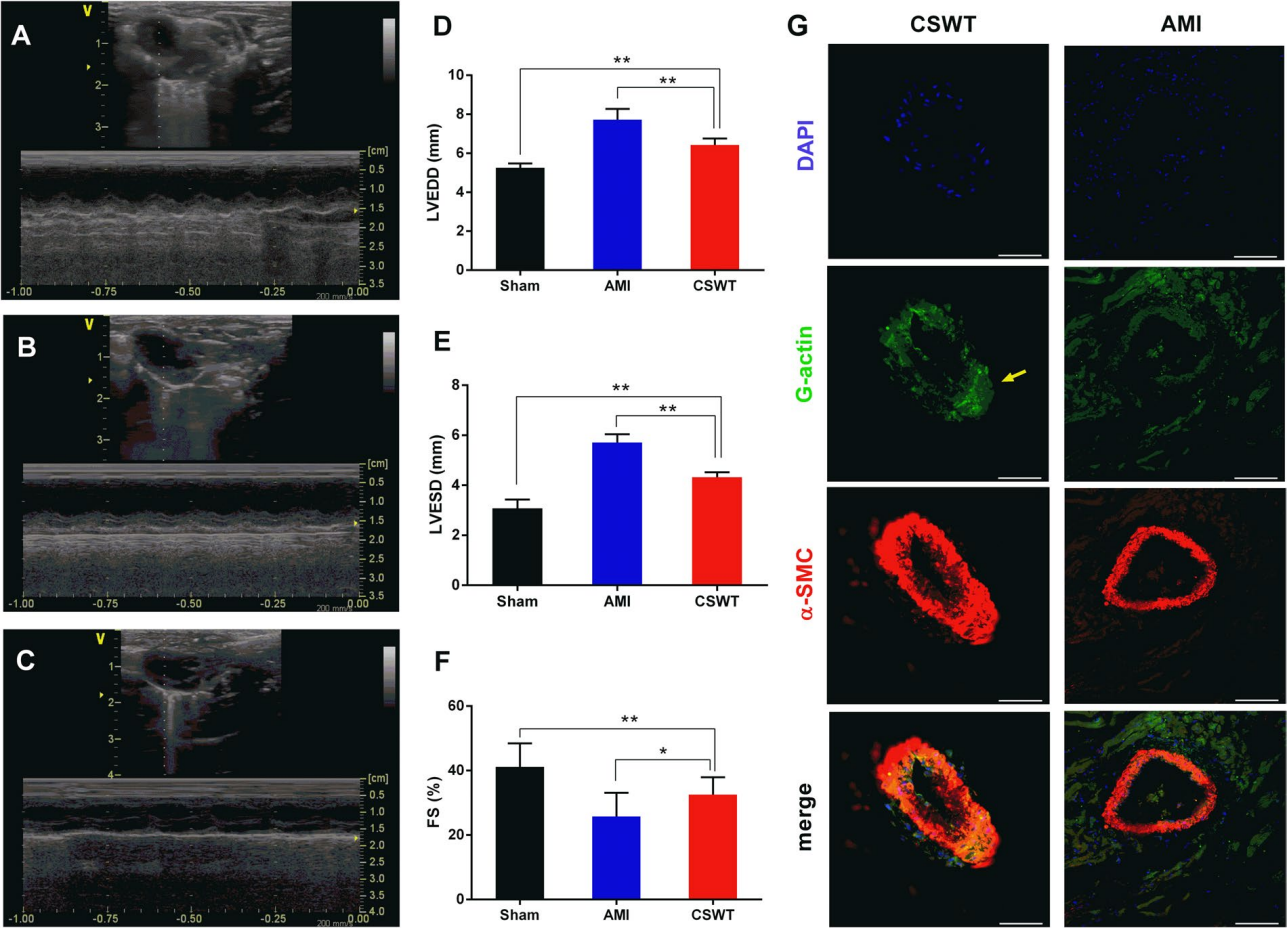
Echocardiography evaluated the cardiac function in rats with AMI. The cardiac function in the Sham group (**A**), the AMI group (**B**) and the CSWT group (**C**) was evaluated. The LVEDD (**D**), LVESD (**E**) (P < 0.01) and FS (**F**) (P < 0.05) and ventricular wall motion were detected in the left ventricular long-axis view. (**G**) coronary collateral vessels were accessed by immunofluorescence confocal microscopy.

Four weeks after LAD ligation, the echocardiographic evaluation showed a considerable improvement of LV wall motion in the CSWT group compared to AMI group (Fig. 1A–C). The LVESD, LVEDD (P < 0.01) and FS (P < 0.05) were markedly improved (Fig. 1D–F) in the CSWT group, yielding a significant difference from the AMI group, which suggested that CWST could alleviate left ventricular remodeling after myocardial infarction.

### Arteriogenesis-related phenotype changes of smooth muscle cells in coronary micrangium

Myocardial sections from the sham group, AMI group and CSWT group were stained by immunofluorescence staining and observed by confocal laser scanning microscope. In the infarction border zone, the thickness of smooth muscle layer was expanded apparently 4 weeks after CSWT (Fig. 1G). A significant increase in the fluorescence intensity of G-actin in the smooth muscle cells of coronary arteriole was also found in the CSWT group compared to the AMI group (Supplementary Fig. S1). These indicated that the smooth muscle cells of the coronary arteriole were transforming from a contractile phenotype to a synthetic phenotype. The differentiation and proliferation of smooth muscle cells was perceived as a sign of arteriogenesis in coronary micrangium. These results suggested that CSWT-induced arteriogenesis effectively contributes to salvaging the myocardium in the infarction border zone and therefore improves LV remodeling characterized by LV enlargement and dysfunction.

### Fibrosis of LV myocardium

**Figure 2:**
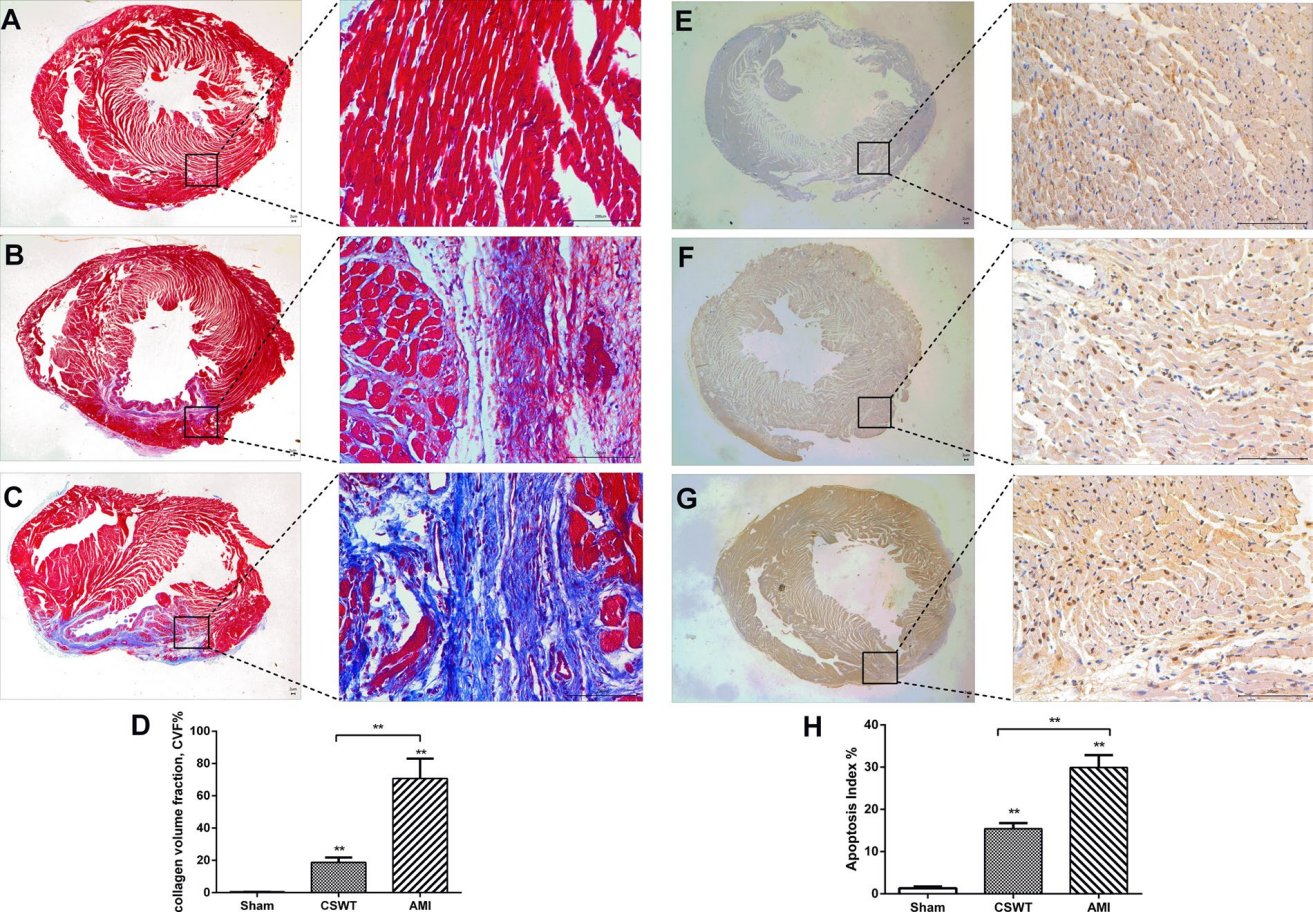
Immunohistochemical staining of fibrosis and apoptotic nuclei in LV myocardium. Masson’s trichrome staining shows remarkably higher fibrotic area following myocardial infarction (**C**) than in the Sham group (**A**) and the CSWT group (**B**). CVF in the three groups (**D**). TUNEL assay shows a notably higher number of apoptotic nuclei after myocardial infarction (**G**) compared with the Sham group (**E**) and the CSWT group (**F**). The apoptotic index of the three groups (**H**). *P < 0.05, **P < 0.01.

Masson staining results showed that compared with the sham and CSWT groups, myocardial fibrosis in the infarction border zone increased obviously in the AMI group 4 weeks after myocardial infarction. There were much more irregularly arranged myocardial fibers and abnormal collagen deposition in the AMI group (Fig. 2A–C). The CVF and the hydroxyproline content in myocardium was higher in the CSWT group than that of the sham group, but was significantly lower compared with that of the AMI group (P < 0.05, Fig. 2D, Supplementary Table S1 and Fig. S2), suggesting that CWST may alleviate fibrosis after myocardial infarction.

### Apoptosis of LV myocardium

TUNEL assay identified notably higher number of apoptotic nuclei in the AMI group and CWST group than that in sham group, and remarkably higher in the AMI group than in the CSWT group (Fig. 2E–G). The cardiomyocyte apoptosis index in the AMI and CSWT groups were markedly higher than that in sham group (29.91 ± 2.96 & 15.42 ± 1.33 vs 1.30 ± 0.41) %, and was significantly lower in the CSWT group compared with that in the AMI group (29.91 ± 2.96 vs 15.42 ± 1.33) %, the differences being statistically significant (P < 0.05, Fig. 2H, Supplementary Table S1). These data suggest that the cardiomyocyte apoptosis after myocardial infarction in SD rats could be alleviated by CSWT application.

**Figure 3:**
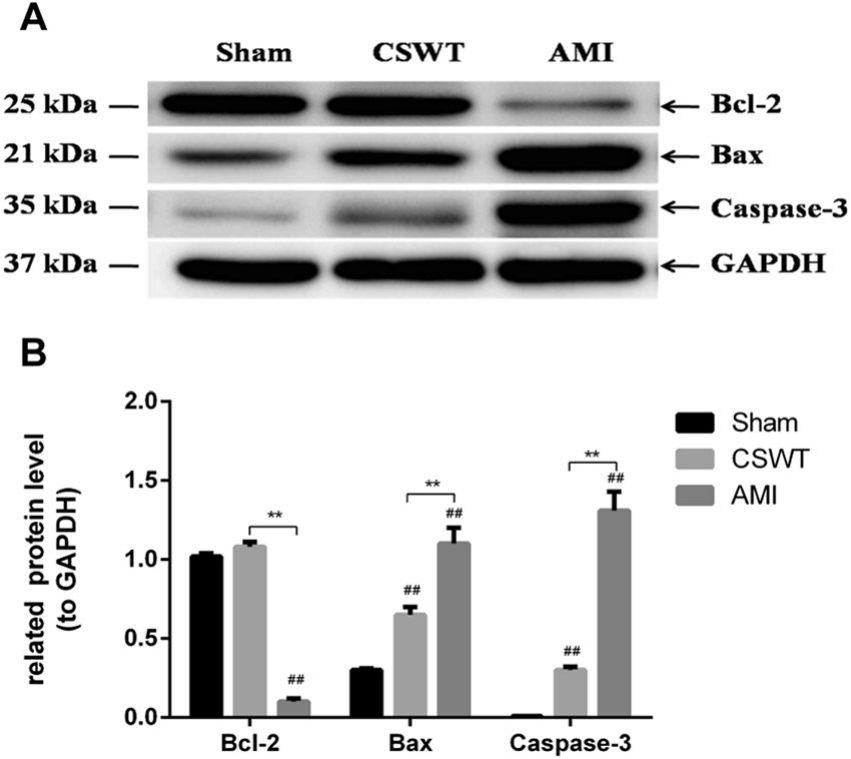
Apoptosis-related proteins were detected by Western blotting. (**A**) Western blot of Bcl-2, Bax and Caspase3 expressions (cropped gels). (**B**) The average relative quantitative expression of Bcl-2, Bax, Caspase-3 abundance in the three groups. n = 5, Mean ± SEM. ^##^*P* < 0.01, compared with Sham. **P < 0.01, comparison between the two groups.

Western blot results revealed that the expression of anti-apoptosis Bcl-2 in the CSWT group and the sham group was higher than that in the AMI group (P < 0.01). In addition, the expression of apoptin Bax in the CSWT group and the AMI group was increased compared with that in the sham group (P < 0.01), while the expression of Bax in the CSWT group was decreased remarkably compared with the AMI group (P < 0.01). The expression of caspase-3 in the CSWT group and the AMI group was increased more than in the sham group (P < 0.01), and caspase-3 expression in the CSWT group was decreased significantly more than in the AMI group (P < 0.01, Fig. 3). All of these suggested that CSWT ameliorates post-MI LV remodeling and cardiac function not only through angiogenesis but also through suppression of cardiomyocyte apoptosis and LV fibrosis.

### Proteomic profiles showed significant differences in protein expression

**Figure 4:**
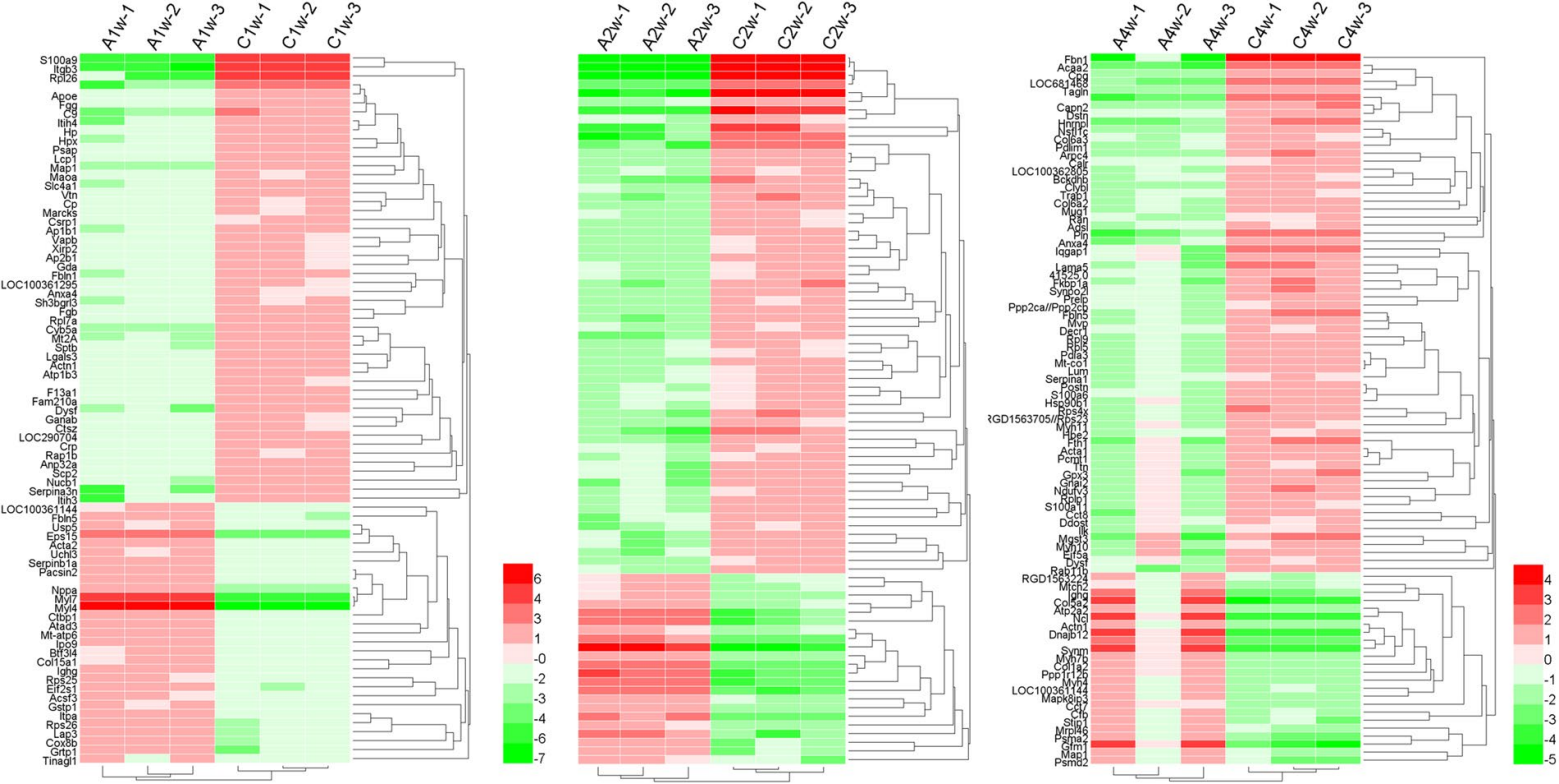
Hierarchical clustering characteristics of differentially abundant proteins in specimens from the AMI group and the CSWT group at the period of 1w, 2w and 4w respectively.

A total of 13553 unique peptides corresponding to 1320 proteins were identified. Among these proteins, 1277, 1193 and 976 proteins were respectively identified in the sample of 1 week (C1 & A1), 2 weeks (C2 & A2) and 4 weeks (C4 & A4), and 922 proteins (69.8%) were observed to be expressed among the three time periods (Supplementary Fig. S3). We used Protein iBAQ values to describe the protein abundance. Proteins with an abundance changing ratio > +/−2.0 and P < 0.05 (t-test) were reserved (including upregulated and downregulated proteins). Following this criterion, a total of 79 proteins in the CSWT group at 1 week (50 up-regulated and 29 down-regulated proteins) were defined as differential abundance proteins, compared with the AMI group. In the CSWT group at 2 weeks, 82 differential abundance proteins (60 up-regulated and 22 down-regulated proteins) were identified. And 89 differential abundance proteins (65 up-regulated and 24 down-regulated proteins) were identified in the CSWT group at 4 weeks. All those proteins are shown in Supplementary Table S2. The hierarchical cluster analysis of differentially expressed proteins, which was identified by feature selection from the pairwise comparison at three separate periods, showed that the number and pattern of molecular changes in the CSWT group was dramatically different from those in the AMI group (Fig. 4).

### Enrichment analysis

**Figure 5:**
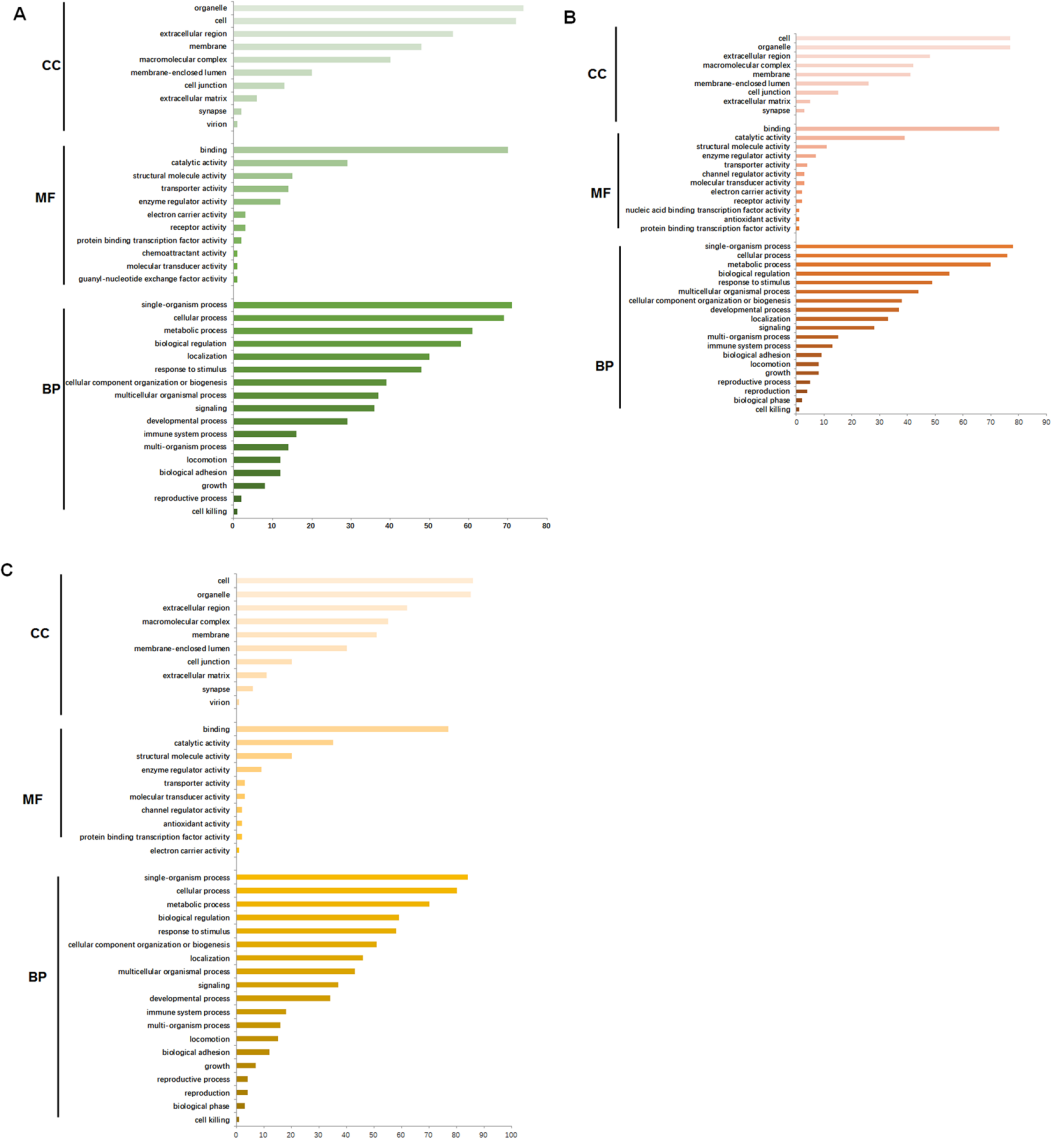
GO analysis of differentially expressed proteins among different biological processes. The GO terms with Biological Process, Molecular Function and Cellular Component of differentially expressed proteins in 1w (**A**), 2w (**B**), 4w (**C**).

To understand the functions and biological processes involved in CWST, differentially expressed proteins of the CWST group for different times were enriched to GO terms, as shown in Fig. 5. At 1 week after CSWT treatment, the most abundant GO terms were: Single-organism process, cellular process and metabolic process (biological process); binding (molecular function); organelle, cell and extracellular region (cellular component) (Fig. 5A). At 2 weeks after CSWT treatment, the most abundant GO terms were: Single-organism process, cellular process and metabolic process (biological process); binding (molecular function); organelle, cell and extracellular region (cellular component) (Fig. 5B). At 4 weeks after CSWT treatment, the most abundant GO terms were: Single-organism process, cellular process and metabolic process (biological process); binding (molecular function); organelle, cell and extracellular region (cellular component) (Fig. 5C). In summary, single-organism process and cellular process were identified as the primary biological process that the differentially expressed proteins were involved in. Binding was the major molecular function. The differentially expressed proteins were distributed mainly in the cell and organelle.

**Figure 6:**
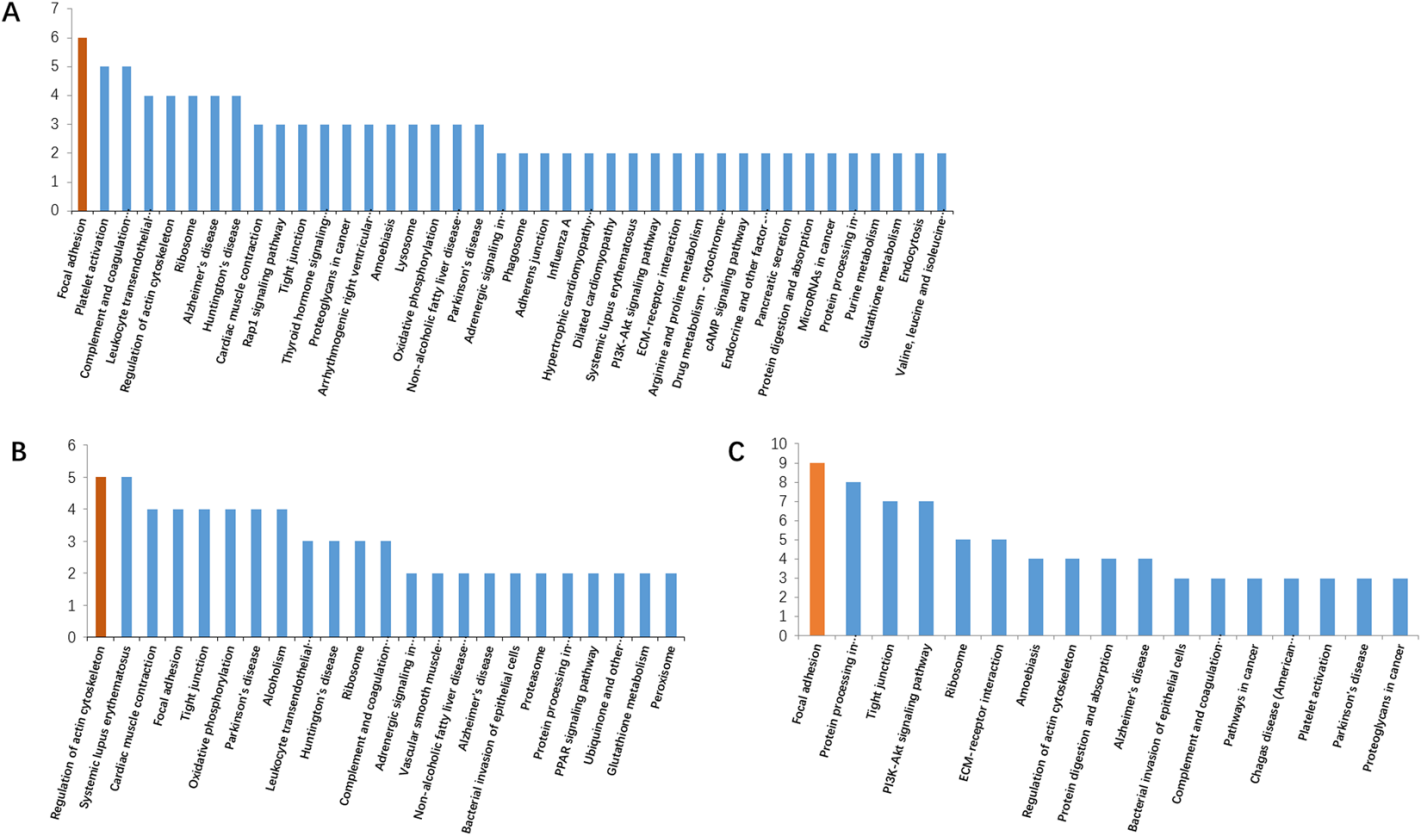
KEGG pathway enrichment analysis of differentially expressed proteins.

**Figure 7:**
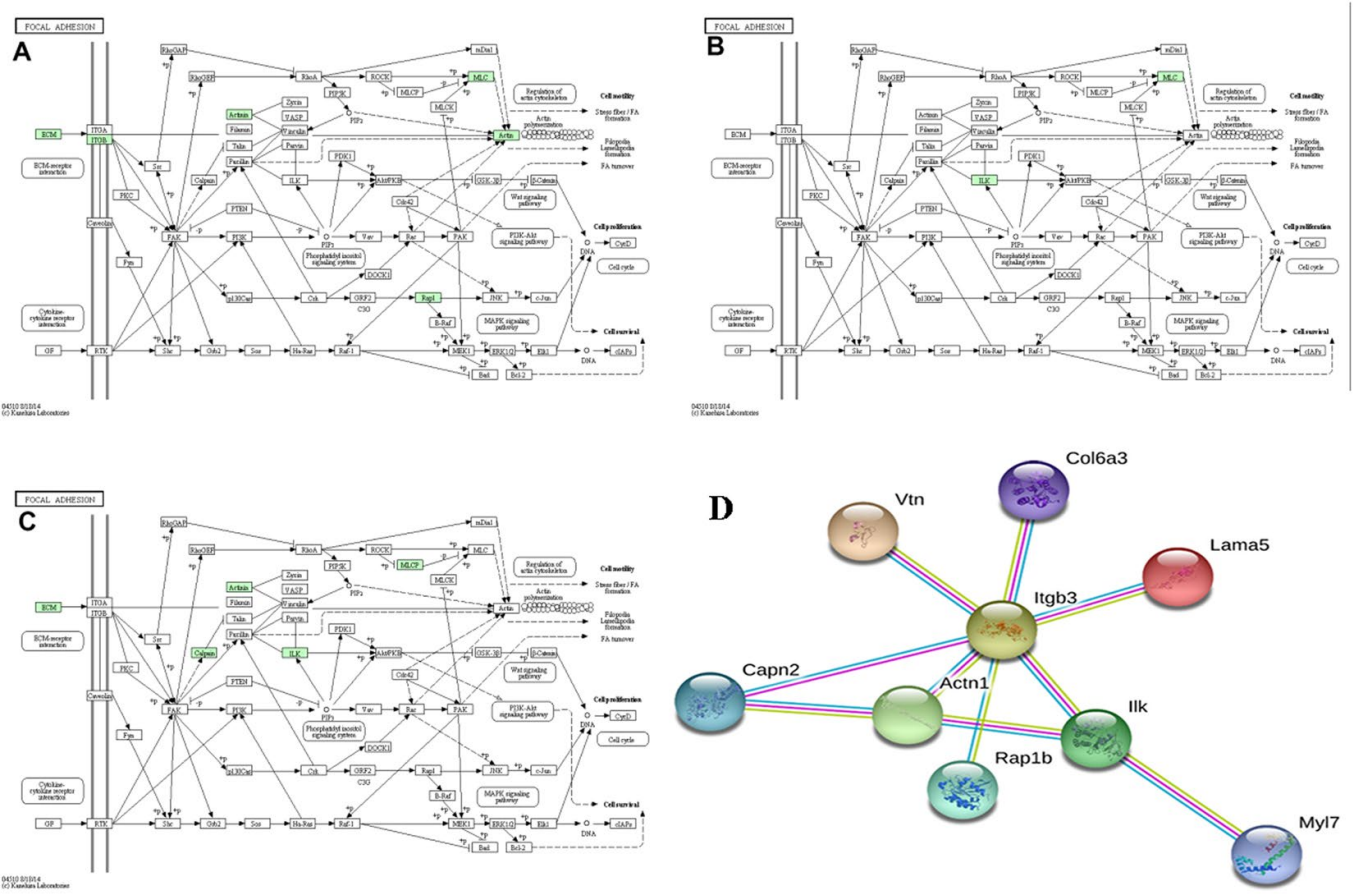
Focal adhesion pathway of the CSWT group at 1w (**A**), 2w (**B**), 4w (**C**)^15–17^. (**D**) Protein-protein interactions of the 9 differentially expressed proteins in the focal adhesion pathway.

In cells, different proteins interact with each other and complete a series of biochemical reactions in order to perform their biological functions. Therefore, KAAS (KEGG Automatic Annotation Server) was used to uncover the main metabolism and signaling pathways underlying the differentially expressed proteins, and the identified proteins were searched against the rat protein sequences in the KEGG GENES database. As a result: ① C1-A1: 43 differentially expressed proteins were mapped in 108 signaling or metabolic KEGG pathways; ② C2-A2: 46 differentially expressed proteins were mapped in 66 signaling or metabolic KEGG pathways; ③ C4-A4: 52 differentially expressed proteins were mapped in 107 signaling or metabolic KEGG pathways. Most differentially expressed proteins were enriched in focal adhesion, protein processing in endoplasmic reticulum, tight junction, PI3K-Akt signaling pathway, ECM-receptor interaction, platelet activation, complement and coagulation cascades, leukocyte transendothelial migration, regulation of actin cytoskeleton and cardiac muscle contraction (Fig. 6). Focal adhesion signaling pathways was well enriched by significantly changing proteins during all the time of CSWT treatment (Fig. 7A–C)^15–17^. Among the proteins related to the Focal adhesion signaling pathways, four proteins were up-regulated, including Integrin-linked protein kinase (ILK), Collagen α-3(VI), laminin α5 and Capn2. Five proteins were down-regulated, including Rap1b, Vitronectin, Integrin β3, α-actinin-1 and Myl7. These annotations provide a valuable resource for investigating specific processes, functions and pathways in the application of CSWT.

### Protein-protein interaction network

**Figure 8:**
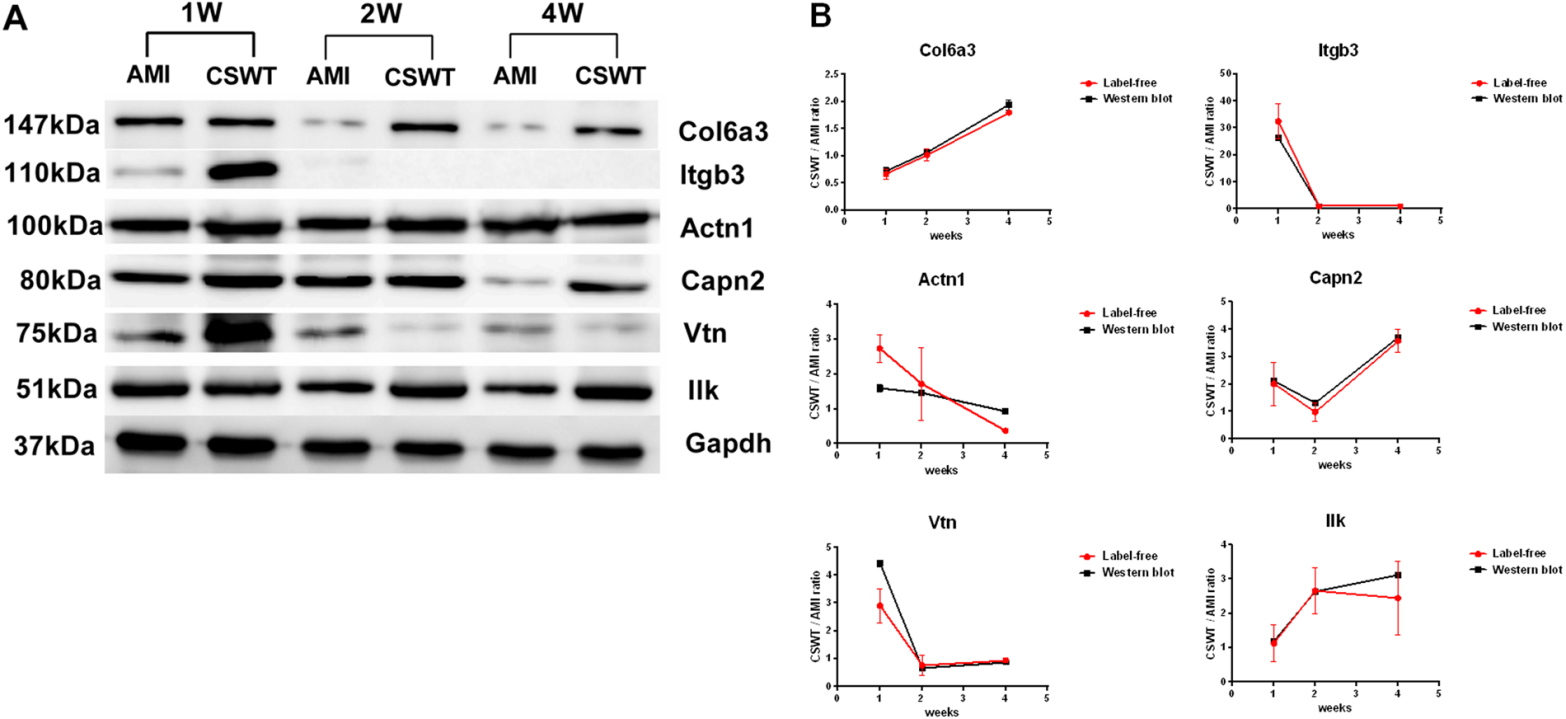
Validation of the 6 protein levels (n = 3 for each time point). (**A**) Western blotting of Col6a3, Itgb3, Actn1, Capn2, Vtn, Ilk and GAPDH (cropped gels). (**B**) Graphical representations of Col6a3, Itgb3, Actn1, Capn2, Vtn, ILK levels in Western blotting and label-free quantative proteomic results.

In addition to KEGG, the nine proteins differentially expressed were also input into the STRING database to construct a protein-protein interaction network. The results showed that among the nine differentially expressed proteins related to focal adhesion signaling pathways, ILK and Itgb3 are located in the center of the interaction network (Fig. 7D), suggesting that they play a central role in the signal pathway. Western blot confirmed that the time-varying expression trend of ILK, Collagen α-3(VI), Capn2, Vitronectin, integrin 3 and α-actinin-1 were consistent with the results of Label free (Table 1, Fig. 8).

**Table 1:** Differentially expressed proteins enriched in Focal adhesion pathway.

Gene names	Col6a3	Capn2	Vtn	Actn1	Itgb3	Ilk
Protein names	Collagen alpha-3(VI) chain	Calpain-2 catalytic subunit	Vitronectin	Alpha-actinin-1	Integrin beta-3	Integrin-linked protein kinase
Protein IDs	D4A111	Q07009; Q6LBQ2	Q7TQ11; Q3KR94; Q62905; CON__Q3ZBS7	Q6T487; Q9Z1P2; Q6GMN8	Q8R2H2; M0RCF3	Q99J82
**iBAQ (CSWT/AMI)**						
1W-1	40759000/52186000	4374600/1656600	52337000/14695000	164120000/51308000	2139700/0	9144500/7589300
1W-2	35747000/59531000	5239900/2297500	50242000/18181000	141010000/55462000	3259400/124790	6002500/10718000
1W-3	38808000/60564000	4930700/4390800	55742000/23502000	139620000/56089000	2204700/31634	6128500/3769600
**iBAQ (CSWT/AMI)**						
2W-1	43360000/44513000	3458200/3043500	10093000/9671900	136060000/46313000	0/0	5571700/2973700
2W-2	40350000/43113000	3587200/2883000	7767900/20594000	50113000/44046000	0/0	8253000/3046100
2W-3	46705000/40605000	1208400/2120600	11425000/12918000	49226000/45039000	0/79186	8396200/2465800
**iBAQ (CSWT/AMI)**						
4W-1	44616000/24496000	2909800/914860	7816700/8991900	60842000/166820000	0/0	3933800/1211600
4W-2	44906000/25211000	3483600/988710	7105700/6956500	63170000/168660000	0/0	4058800/3284500
4W-3	42133000/23058000	3998700/0	6218400/6514800	64235000/175620000	0/0	5825500/2038300
one-way ANOVA AMI P value	2.53E-06	0.0117297	0.00732792	0.00151024	0.0701251	0.0530856
one-way ANOVA CSWT P value	7.52E-05	0.0633402	5.14E-09	0.140331	1.74E-05	0.00736721

## Discussion

The coronary collateral circulation is critically important as an adaptation of the heart to prevent the damage from ischemic insults. Reconstructing collateral circulation by arteriogenesis is an effective procedure to improve coronary microcirculation. Previous clinical studies have shown that myocardial perfusion and metabolism, angina pectoris symptom, cardiac functions and 6-min walking distance were improved significantly in patients with CSWT treatment^18,19^. Newly published data of a 6-year follow-up demonstrated that the ventricular wall motion, myocardial perfusion, nitroglycerin dosage, New York Heart Association functional class, Seattle Angina Questionnaire score, Canadian Cardiovascular Society class of 38 patients with CSWT treatment were obviously ameliorated than pretherapy^20^. It has been found that CSWT could inhibit the expression of metal matrix proteases (MMPs) in infarcted myocardium, regulate the metabolism of MMPs/TIMP1, stabilize the metabolism of cardiac extracellular matrix, and induce neocapillary formation in the infarction border zone. In addition, CSWT improved myocardial micro-vascular circulation after acute myocardial infarction at early stage in pigs^21^. The improved Rentrop scores of collaterals development indicate that CSWT may take effect through developing of collateral circulation and increasing myocardial perfusion, and then effectively improving the symptoms of angina pectoris, myocardial ischemia and left heart function. However, the biomechanical effect of CSWT on coronary microvascular and myocardium has not been well understood.

CSWT could exert “cavitation effect”, which induces lots of bubbles in myocardium tissue. And these bubbles could enlarge and collapse, and generate a physical force as “shear stress” on cell membranes^22^. It has been found that fluid shear stress (FSS) is an important trigger of arteriogenesis after arterial occlusion or severe stenosis^14,23^. In the natural state, however, FSS will decrease prematurely along with the opening of collateral circulation and collateral arteriogenesis will suspend quickly either. Therefore, increasing the strength and function time of shear stress could be an effective way to solve this problem^24^. CSWT could make constant and steady shock wave into the ischemic myocardium and produces biological effects on vascular endothelial cells and smooth muscle cells directly.

CSWT can upregulate endogenous angiogenic factors and their receptors, and promote endothelial cell proliferation, differentiation, development of local capillaries. Not only that, in this study, in the infarction border zone, the thickness of smooth muscle layer were expanded apparently 4 weeks after CSWT. The up-regulated expression of G-actin in arterioles indicates that SMCs has lost their contractile phenotype and assumed a synthetic and proliferative phenotype. The phenotype changing of SMCs separates arteriogenesis from angiogenesis^25^. It means CSWT could improve myocardial microcirculation by arteriogenesis.

Myocardium in the infarcted area can exhibit necrosis and apoptosis under the stimulation of ischemia and hypoxia after myocardial infarction, which is a major cause of decreased systolic function and early left ventricular remodeling^26^. Nevertheless, cardiomyocyte apoptosis in the border area of the infarcted myocardium and in the non-infarcted area is the main cause of terminal heart failure and LV remodeling^27^. Cardiomyocyte apoptosis activates the expression of apoptosis-related genes through death receptor pathway and mitochondrial pathway. Yu *et al*.^28^ induced apoptosis of H9c2 cells under hypoxic conditions *in vitro* and then implemented CSWT treatment. The results showed that H9c2 cell apoptosis was significantly decreased (enhanced bcl-2 expression, down-regulated Bax and Caspase3 expression). In a model of porcine acute myocardial infarction, the mRNA level of bcl-2 was up-regulated and Bax and Caspase3 were down-regulated in the CSWT group, as well as there being a decrease in the expression of mitochondrial oxidative stress associated proteins^29^. In our experiments, the anti-fibrotic effects of CSWT were closely related to the suppression of apoptosis, and thus alleviated left ventricular remodeling and improved the long-term prognosis of patients.

Recently, scholars have conducted a lot of work on the use of CSWT in the treatment of myocardial ischemia. These studies found that CSWT could improve the clinical symptoms of patients with myocardial ischemia via promoting the expression of related growth factors, and inducing angiogenesis and apoptosis inhibition. However, the specific action mechanism of CSWT has not been clearly explained. In our study, label-free quantitative proteomic technology and bioinformatics analysis method were applied to study the biological processes of CSWT treatment on cardiomyocyte and coronary micrangium using AMI model in rats. We found that the differentially expressed proteins from the three time points were significantly enriched in the focal adhesion signal pathway after myocardial infarction with or without CSWT treatment. The focal adhesion signal pathway could play an important function in the biological process of CSWT acting on the infarcted myocardium. Focal adhesion is the contact point between cells and the surroundings and is also a special device for transmitting mechanical signals to cells^30,31^. The focal adhesion signal pathway regulates a variety of downstream signaling pathways inside and outside cells, and participates in many physiological and pathological processes^32^.

Cells adhere to the extracellular matrix (ECM) via integrin-mediated adhesion that links the ECM to the actin cytoskeleton which determines how cells respond to mechanical forces, humoral factors and various developmental signals. Transmembrane integrin molecules, which serve as receptors for ECM proteins, are associated, through their cytoplasmic domains, with a complex of proteins including ILK, focal adhesion kinase (FAK), talin, α-actinin, and vinculin and many others^33,34^. It has been found that ILK, a mechanoreceptor protein, regulates SERCA-2a and PLN to improve the mechanical forces conduction of damaged cardiomyocyte in patients with dilated cardiomyopathy, and eventually ameliorating the myocardial systolic function^35^. Meanwhile, ILK-PINCH-parvin complexes regulate PKB/Akt activity and enhance myocardial contractility during heart failure^36^. In addition, bone marrow mesenchymal stem cells (BMSCs) with ILK transfection via intracoronary-administered can inhibit myocardial collagen synthesis and proliferation of fibroblast after myocardial infarction in pigs^37^, at the same time increasing the survival rate, proliferation, differentiation and angiogenesis of MSCs transplantation, significantly improving the myocardial function^38^. In any case, the role of ILK in the cardiovascular system is extensive and complex. In the course of our studies, ILK in the differential expression proteins-enriched focal adhesion signaling pathway continued to show an upward tendency during the treatment cycle of CSWT. Studies have shown that ILK plays an important role in the phenotypic changes of vascular smooth muscle cells^39^. Therefore, the time-varying expression of ILK may be one of the key regulators of coronary angiogenesis promoted by CSWT.

In conclusion, we demonstrated that CSWT alleviates post-MI left ventricular remodeling and improves cardiac function in rats *in vivo*, which is associated with the angiogenic effects and the antiapoptotic effects, thus describing a novel aspect of the therapy for AMI. Having demonstrated a positive effect of CSWT in the present study, we further sought to investigate the precise mechanism and gain greater understanding of the angiogenic and antiapoptotic effects of shock waves. During the process of CSWT’s mechanical signal transforming into a biological effect on the heart of AMI rats, the focal adhesion signaling pathway may have a central role in the related signal network. ILK and other sequential differentially expressed proteins were closely related to the phenotype of coronary micrangium arteriogenesis with CSWT therapy. These proteins would be the key factors that account for a series of biomechanical effects and are expected to become the new breakthrough point for biomechanical mechanism research into CSWT. This study indicated that CSWT application is a potentially feasible, noninvasive, clinically relevant approach to protecting cardiomyocyte against ischemic apoptosis and necrosis, comparable to invasive, surgical interventions, which carry relatively higher risks.

## Methods

The investigation conforms to the Guide for the Care and Use of Laboratory Animals established by the US National Institutes of Health (2011–2012). The present study was approved by the Institutional Committee for Use and Care of Laboratory Animals at Yan’an Affiliated Hospital of Kunming Medical University.

All procedures were performed in accordance with the Institutional Guidelines for Animal Research and the investigation conformed to the Guidelines for Care and Use of Laboratory Animals published by the US National Institutes of Health (NIH Publication No. 85–23, revised 2011).

### SD rats model of acute myocardial infarction

A total of 70 adult male SD rats (240 to 288 g in body weight) were used in this study: AMI group (n = 30), CSWT group (n = 30) and sham group (n = 10). A rat model of acute myocardial infarction through ligation of the left anterior descending artery was performed in both AMI group and CSWT group. Rats were anesthetized with isoflurane inhalation and ventilated by tracheal intubation. An oblique incision was made along the left sternal border. After Inserting chest retractor, the chest cavity was opened. The position for the ligature of the main descending left ventricle coronary artery is approximately 2 mm lower than the tip of the left auricle. Confirm the occlusion of LAD by checking for appearance of a paler color and abnormal motion in the anterior wall of the LV that should appear within a few seconds after ligation. And those in the sham group were only given thoracotomy. The rats in the AMI group were separately sacrificed at 1, 2 and 4 weeks after MI. Similarly, the rats in the CSWT group were respectively harvested at 1, 2 and 4 weeks after CSWT treatment. They were labeled as CSWT group (subgroup C1, C2, and C4) and AMI group (subgroup A1, A2 and A4), n = 10. Rats in the sham group were sacrificed at 4 weeks.

### CSWT treatment

Animals in the CSWT group were anaesthetized with inhaled isoflurane and became unresponsive to a moderate pain stimulus while still normally breathing spontaneously. CSWT treatment was performed with the MODULITH SLC SW therapy device (Storz Medical, Switzerland). Shocks waves pulse were focused on the ischemic myocardium. A total of 200 shots were delivered at an energy flux density of 0.24mJ/mm^2^ during every treatment session (60 shots per minutes). The CSWT treatment was began on the second day after LAD ligation surgery and was sustained for 4 weeks, with three sessions in 1 week^40^. Animals in the AMI and sham groups received the same anesthesia procedures but without the CSWT.

### Echocardiographic evaluation

High-frequency echocardiography was used to evaluate cardiac function 4 weeks after surgery. General Electric (GE) Vivid 7/E9 color Doppler ultrasonography system with a 10 MHz transducer was performed to observe the LV wall motion and more than five dynamic images of the cardiac cycle were taken and stored on CD. Left ventricular end-systolic diameter (LVESD) and left ventricular end-diastolic dimension (LVEDD) were measured. Fraction shortening (FS) = (LVEDD − LVESD)/LVEDD × 100%. All ultrasound measurements were taken from the mean values of three cardiac cycles^40^.

### Methods for myocardial sample preparation

We took the myocardium in the infarction border zone (~2 mm in width around the infarcted myocardium) as specimens for the following experiments. One part of the myocardium specimens were freshly frozen in liquid nitrogen and stored in −80 °C refrigerator. Another part of the myocardium specimens were embedded in paraffin after fixed in paraformaldehyde for 24 h. The embedded myocardium specimens were sliced into serial 5-μm-thick sections and placed on poly-L-lysine-coated glass slides^41^.

### Masson’s trichrome staining

The myocardium fibrosis in the infarction border zone was detected by Masson’s trichrome staining according to the protocol. The fibrosis areas in five random fields of the infarction border zone in each heart (200 magnifications) were evaluated using software Image-pro Plus 6.0 (Media Cybernetics Inc., Bethesda, Maryland, USA). The extent of fibrosis was quantified as the collagen volume fraction (CVF) = (collagen area/myocardial area) × 100%^42^.

Hydroxyproline concentration in the myocardium was determined using a commercialization Hydroxyproline assay kit according to the manufacture’s instruction (Cell Biolabs, Inc., San Diego, CA).

### Immunofluorescence confocal microscopy

Immunofluorescence confocal microscopy was used to observe the arteriogenesis of the coronary micrangium. Tissue sections were incubated overnight at 4 °C with first primary monoclonal antibodies against G-actin (1:100, rabbit polyclonal) and α-SMA (1:100, rabbit polyclonal, both from Abcam Inc., Cambridge, Massachusetts, USA). The fluorescein isothiocyanate (FITC)-labeled antibody (1:100, KPL, USA) and *Cy*^TM^3-labeled antibody (1:100, KPL, USA) were incubated for 30 min at room temperature. Nuclei were stained with DAPI (Boster Biotechnology Co. Ltd, Wuhan, China). Controls were performed with/without the primary antibody or with/without the secondary antibody on the three group tissues, and were included in all experiments to correct for background fluorescence. For fluorescence analyses, an Olympus FV1000 confocal microscope (Olympus Tokyo, Japan) was used^43^.

### TUNEL

The apoptosis-related proteins were evaluated by TUNEL using the *In Situ* Cell Death Detection Kit (Boehringer Mannheim, Indianapolis, IN) according to instructions. The percentage of apoptotic cells was calculated by counting 200 cells in 5 fields in each experiment. Five high magnification fields were randomly selected from the border zone of infarcted myocardium in each section and the number of positive cells was recorded. Apoptosis Index (AI%) = Apoptotic cell nuclei / total cell nuclei × 100%^44^.

### Western blot

The extracted samples were subjected to SDS-PAGE by using specific antibody for Bcl2 (1:1000), Bax (1:1000), Caspase3 (1:1000), GAPDH (1:1000) (Millipore, Germany). The bands were detected with an EZ-ECL kit (BI Biological Industries, 20-500-120) in Imagequant LAS4000 mini (GE Healthcare) after incubation with secondary antibodies. Plus Image-Pro 6.0 software was used for image analysis. GAPDH was used as control for normalization^40^.

### Samples preparation and LC-MS/MS label-free quantitative proteomics

Whole myocardial tissues preparation and protein extraction of were carried out by a standard protocol^45^. Briefly, tissues were cut into pieces and rinsed on ice with PBS until no blood could be observed. Then 1 mL HU buffer (20 mmol/L HEPES, 9 mol/L Urea, pH = 8.0) was added into each sample and the mixture was homogenized by grinding. After that, the samples were homogenized on ice by ultrasonication (80 W, 10 times for 15 s, each time at 15 s intervals). The homogenized samples were then centrifuged at 13400 rpm for 30 min and the supernatant was collected and quantified by BCA protein assay kit (Biorad procedure, USA). A total of 4 μg of each protein sample was subjected to SDS-PAGE and Coomassie blue staining to determine the protein cleavage (Supplementary Fig. S4). According to the quantitative results, DTT were added into 200 μg of proteins to a final concentration of 10 mmol/L and left at 37 °C for 1 h. Then, the protein samples were alkylated with 20 mM iodoacetamide (final concentration). After incubation at room temperature for 30 min protected from light, 2 μg Lysc were added into samples and incubated at 37 °C for 3 h. The samples were diluted with 50 mmol/L chondroitinase ABC to a final concentration of 20 mmol/L HEPES and 1 mol/L Urea. Finally, 4 μg of trypsin (Promega) was added and mixed for 1 min at 600 rpm. The sample mixture was allowed to digest at 37 °C for 16–18 h. The reaction was terminated with 0.25% trifluoroacetic acid (TFA). After desalting with C18 Cartridge, the resulting samples were collected and the peptide content was analyzed at OD280^46^.

MS experiments were performed on a Q Exactive mass spectrometer (Thermo Scientific, USA) equipped with EASY-nLC1000 (Thermo Scientific, USA). The HPLC mobile phase was composed of buffer A (0.1% formic acid) and buffer B (0.1% formic acid and 84% acetonitrile), with a linear gradient of buffer B as follows: 0–45% for 0–100 min; 45–100% for 100–108 min; 100% for 108–120 min. Peptide mixture (4 μg) was loaded onto a Thermo EASY column (150 μm * 20 mm, RP-C18), and separated with the Thermo EASY column (150 μm * 100 mm, RP-C18) at 300 nL/min^46^.

The mass spectrometer data were analyzed by the Q-Exactive mass spectrometer (Thermo Finnigan, USA) in the positive ion mode. A precursor ion scan over the m/z range 300–1800 (resolution 70,000 at m/z = 200) was followed by MS scans of the 20 most abundant ions upon fragmentation in the HCD cell at normalized collision energy (resolution 17,500 at m/z = 200).

The raw LC-MS/MS data from all samples were processed by MaxQuant software (version: 1.3.0.5) for label-free relative quantification analysis. Database was the UniProt database (uniprot_rat_34151_20150114.fasta, downloaded 14 Jan 2015, with 34151 total entries). The Maxquant parameters were uploaded in Supplementary Table S3. The main search was set at a precursor mass window of 6 ppm. The search followed an enzymatic cleavage rule of Trypsin/P with allowance for two missed cleavage sites and a mass tolerance of 20 ppm for fragment ions. The downstream analysis was conducted by Perseus software (version 1.3.0.4). Absolute protein quantitation was performed using the intensity-based absolute quantification (iBAQ) method^47^. A false discovery rate (FDR) of up to 1% was allowed for peptide spectrum match and protein assembly, and the FDR was estimated using the reversed peptide sequences.

The subcellular location of each identified protein was predicted based on the GeneOntology annotation (www.geneontology.org). Pathways enriched with significantly changing proteins were determined using pathway mapping tool based on the KEGG (Kyoto Encyclopedia of Genes and Genomes) pathway database (http://www.genome.jp/kegg/pathway.html).

### Statistical analysis

All numerical data are presented as mean ± S.E.M. Statistical differences between two groups were evaluated with Independent Samples t-test. Proteins up-regulated or downregulated in the CSWT groups (C1/A1, C2/A2, and C4/A4) were extracted using the following parameters: max fold change > 2.0 (upregulation) or fold changes < 0.5 (downregulation); p < 0.05. One-way ANOVA analysis was conducted to compare protein abundance changes among different times (C1, C2 and C4; A1, A2 and A4). A value of P < 0.05 was considered to be statistically significant. Statistical chart was designed with GraphPad Prism 6.0 (San Diego, CA, USA)^43^.

### Data availability

The datasets generated and/or analyzed during the current study are available from the corresponding author on reasonable request.

## Electronic supplementary material

Supplementary information
