# Supplementary material for: Cardiac shock wave therapy promotes arteriogenesis of coronary micrangium, and ILK is involved in the biomechanical effects by proteomic analysis
Source: Sci Rep. 2018 Jan 29;8:1814. doi: 10.1038/s41598-018-19393-z (PMC5788936; doi:10.1038/s41598-018-19393-z)
Supplement: Supplementary file 1 — Supplementary information [file 41598_2018_19393_MOESM1_ESM.pdf]

Cardiac shock wave therapy promotes arteriogenesis of coronary micrangium, and ILK is involved in the biomechanical effects by proteomic analysis

Wenhui Yang[1,2], Yan He[1,2], Lulu Gan[1,2], Fan Zhang[1,2], Baotong Hua[3], Ping Yang[3], Juan Liu[4], Li Yang[1,2]*, Tao Guo[3]*

Supplementary Fig. S1 The quantified analysis of fluorescence intensity of G-actin

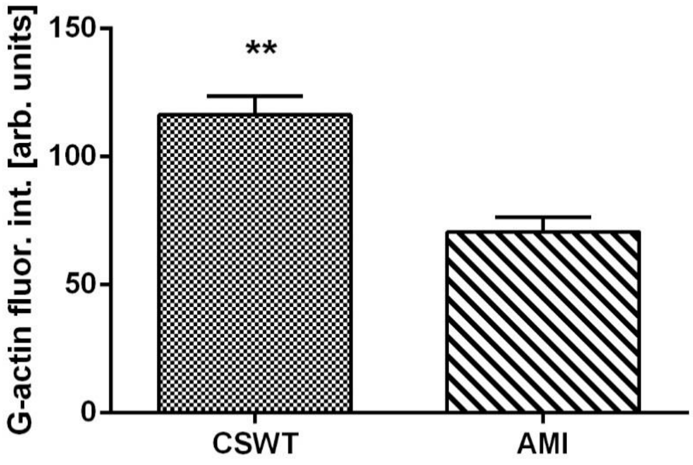

Supplementary Fig. S2 The hydroxyproline assay for quantification of fibrosis

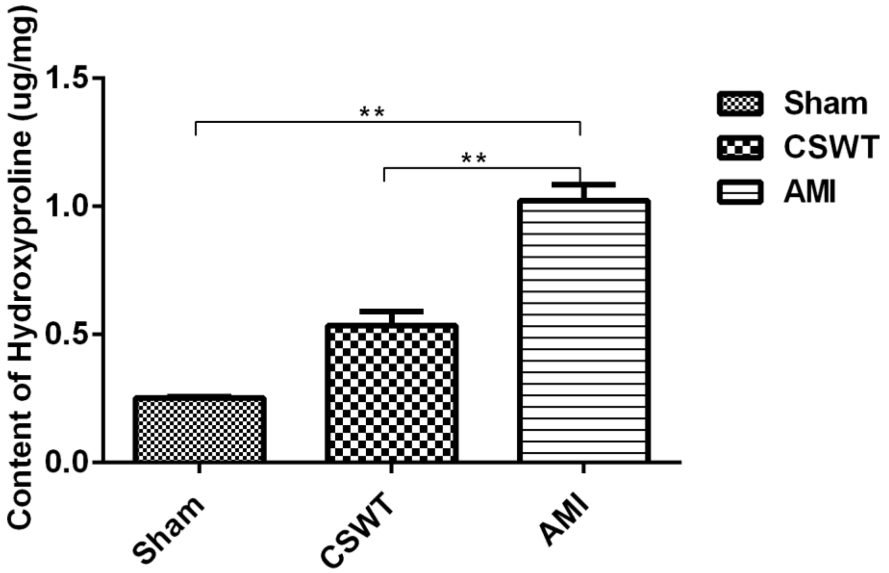

Supplementary Fig. S3 Venn diagram shows the overlap of the identified proteins in the three different periods

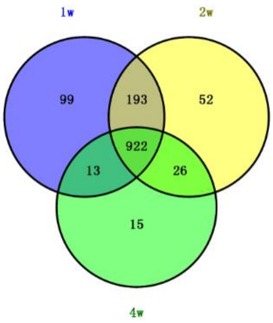

Supplementary Fig. S4 SDS-PAGE stained with Coomassie brilliant blue. The result indicated that proteins were extracted successfully.

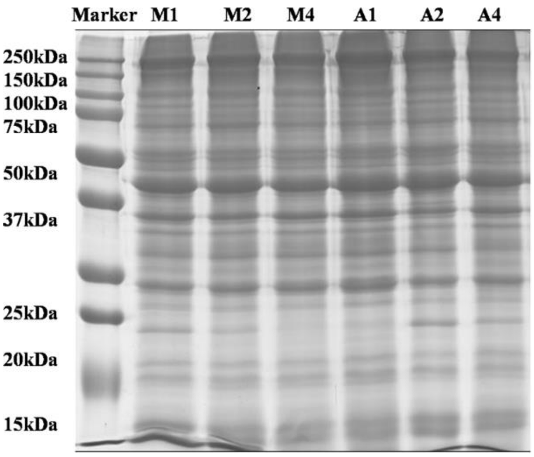

Supplementary Table S1 The CVF and AI of myocardium（ $\bar{x}\pm s$）

	N=10	CVF(%)	Hydroxyproline (ug/mg)	AI
Sham	N=10	0.31± 0.17	0.25±0.009	1.30±0.41
AMI	N=10	70.70±12.34**	1.002±0.152**	29.91±2.96**
CSWT	N=10	18.70±13.06$^{**\#\#}$	0.53±0.132$^{**\#\#}$	15.42±1.33$^{**\#\#}$

Note： ** Compared with Sham group, $P<0.01$. $^{\#\#}$ Compared with AMI group, $P<0.01$.

Supplementary Table S2 The quantitative results of proteins

Comparisons	Up-	Down-	All-
C/A1	50	29	79
C/A2	60	22	82
C/A4	65	24	89

Up-： up-regulated differentially expressed proteins； Down-： down-regulated differentially expressed proteins； All： All the differentially expressed proteins.

Supplementary Table S3 Maxquant parameters

Item	Value
Enzyme	Trypsin
Max Missed Cleavages	2
Main search	6 ppm
First search	20 ppm
MS/MS Tolerance	20 ppm
Fixed modifications	Carbamidomethyl (C)
Variable modifications	Oxidation (M) , Acetyl (Protein N-term)
Database	uniprot_rat_34151_20150114.fasta
Database pattern	Target-Reverse
Include contaminants	True

Peptide FDR	≤0.01
Protein FDR	≤0.01
Time window (match between runs)	2min
Protein quantification	Razor and unique peptides were used for protein quantification.
iBAQ	True
